# Hepatitis E virus seroepidemiology: a post-earthquake study among blood donors in Nepal

**DOI:** 10.1186/s12879-016-2043-8

**Published:** 2016-11-25

**Authors:** Ashish C. Shrestha, Robert L. P. Flower, Clive R. Seed, Manita Rajkarnikar, Shrawan K. Shrestha, Uru Thapa, Veronica C. Hoad, Helen M. Faddy

**Affiliations:** 1 Research and Development, Australian Red Cross Blood Service, 44 Musk Avenue, Kelvin Grove, Brisbane, QLD 4059 Australia; 2 School of Medicine, The University of Queensland, 288 Herston Road, Herston, Brisbane, QLD 4006 Australia; 3 Medical Services, Australian Red Cross Blood Service, Herdsman, Perth, WA 6017 Australia; 4 Central Blood Transfusion Services, Nepal Red Cross Society, Kathmandu, Nepal

**Keywords:** Blood donor, Earthquake, Hepatitis E virus, Hepatitis, Jaundice

## Abstract

**Background:**

As one of the causative agents of viral hepatitis, hepatitis E virus (HEV) has gained public health attention globally. HEV epidemics occur in developing countries, associated with faecal contamination of water and poor sanitation. In industrialised nations, HEV infections are associated with travel to countries endemic for HEV, however, autochthonous infections, mainly through zoonotic transmission, are increasingly being reported. HEV can also be transmitted by blood transfusion. Nepal has experienced a number of HEV outbreaks, and recent earthquakes resulted in predictions raising the risk of an HEV outbreak to very high. This study aimed to measure HEV exposure in Nepalese blood donors after large earthquakes.

**Methods:**

Samples (*n* = 1,845) were collected from blood donors from Kathmandu, Chitwan, Bhaktapur and Kavre. Demographic details, including age and sex along with possible risk factors associated with HEV exposure were collected via a study-specific questionnaire. Samples were tested for HEV IgM, IgG and antigen. The proportion of donors positive for HEV IgM or IgG was calculated overall, and for each of the variables studied. Chi square and regression analyses were performed to identify factors associated with HEV exposure.

**Results:**

Of the donors residing in earthquake affected regions (Kathmandu, Bhaktapur and Kavre), 3.2% (54/1,686; 95% CI 2.7–4.0%) were HEV IgM positive and two donors were positive for HEV antigen. Overall, 41.9% (773/1,845; 95% CI 39.7–44.2%) of donors were HEV IgG positive, with regional variation observed. Higher HEV IgG and IgM prevalence was observed in donors who reported eating pork, likely an indicator of zoonotic transmission. Previous exposure to HEV in Nepalese blood donors is relatively high.

**Conclusion:**

Detection of recent markers of HEV infection in healthy donors suggests recent asymptomatic HEV infection and therefore transfusion-transmission in vulnerable patients is a risk in Nepal. Surprisingly, this study did not provide evidence of a large HEV outbreak following the devastating earthquakes in 2015.

## Background

Hepatitis E virus (HEV) has gained public health attention as one of the causative agents of viral hepatitis. The four genotypes of this non-enveloped RNA virus differ in mode of transmission and geographical occurrence [1, 2]. In developing countries, major outbreaks of HEV with genotypes 1 and 2 are associated with transmission via the faecal-oral route [1, 3]. In developed countries, HEV has traditionally been associated with travel to countries endemic for HEV [4, 5], however, locally acquired HEV infections, associated with zoonotic transmission, are increasingly reported in such countries [5–8].

In Nepal, regular HEV outbreaks have occurred during the previous 4 decades, with reported outbreaks in 1973, 1981–1982, 1987, 1995 and 2014 [9–11]. During these outbreaks, a maternal mortality rate of 21–25% was reported [9]. During an outbreak in Biratnagar in 2014, the case fatality rate was 0.2% [11]. HEV IgG prevalence in 1999–2000 was estimated to be 38% among the general population of Nepal [9]. Kathmandu was designated hyper-endemic for HEV, with rural areas non-endemic [9]. A recent study has demonstrated HEV IgG prevalence of 47% among patients visiting a hospital in Kathmandu [12]. HEV infections have also been reported in travellers to Nepal [13–15]. Poor infrastructure development in terms of water supply and sewerage systems can facilitate the contamination of drinking water [16], especially during the summer monsoon season [17]. Epidemics have been associated with faecal contamination of water, and molecular characterization has shown genotype 1 as a cause of acute HEV infection [18]. HEV antibodies and RNA have been detected in farm swine from Kathmandu [19], indicating the possibility of zoonotic transmission in addition to the usual faeco-oral route.

The recent devastating earthquakes that occurred on 25th April and 12th May 2015 in Nepal raised concerns that the risk of an imminent HEV outbreak was very high, with HEV possibly causing up to 510 deaths in pregnant women [20]. During these earthquakes and their aftermath, 8,891 people lost their lives [21], with many left homeless having to share common shelter under tents for months. Under such overcrowded living conditions poor sanitation and hygiene were likely and individuals no doubt had limited access to safe drinking water, contributing to increased potential for infectious disease outbreaks [20, 22]. The burden of HEV at this time was also expected to be high due to the approaching summer monsoon season and limited access to health facilities [20, 23]. We therefore sought to estimate the rate of previous and recent HEV infection in Nepalese blood donors in the months following the large earthquakes. This study aimed to provide surveillance data about HEV in Nepal, determine the possible impact of the recent earthquakes through serological evidence of recent HEV exposure, and analyse variables as possible risk factors for exposure to HEV.

## Methods

### Sample population

A total of 1,845 blood donors eligible to donate blood as per the criteria of the Central Blood Transfusion Service (CBTS), Nepal Red Cross Society (NRCS) were included in this study. A cross section of samples were collected at blood transfusion services in Kathmandu (*n* = 1,435), Chitwan (*n* = 159), Bhaktapur (*n* = 135), and Kavre (*n* = 116), during the months June-September, 2015. The required sample size for Kathmandu was estimated, using standard methods [24], to be 1,448, based on the assumption of 38% HEV IgG prevalence [9], 95% confidence interval, and an absolute precision of 2.5%. Sample numbers from the other districts were based on accessibility to the donor population.

### Sample collection

Blood donor samples were collected in BD Vacutainer® PPT Plasma Preparation Tubes (Becton, Dickson and Company (BD) Biosciences, San Diego, USA). Samples were centrifuged at 2,500 rpm for 5 min before storage at −20 °C until testing.

### Variables obtained through additional questionnaire

In order to identify possible risk factors associated with HEV exposure, the following variables were included in the additional questionnaire:

1. Donor status: Donors who had previously donated were categorised as repeat donors and those who were donating for the first time as new donors.
2. History of jaundice: Jaundice was defined as any known feature of jaundice including yellow discolouration of skin and hepatitis, whether or not a donor required medical support. Family member jaundice referred to when any family member of a donor had jaundice, as defined above.
3. Source of drinking water: Donors were asked about their drinking water source, whether it be from a community tap, municipality tap, or underground. Community tap refers to a common tap in the community with a reservoir tank in that local area. Municipality tap is treated/purified and is usually supplied to the home by the local government authority. An underground source refers to any ground water source including hand-pumps and wells. Donors responding to two or more options were categorised as relying on multiple sources. Those reporting sources other than those mentioned above including bottled water were categorised as ‘other’.
4. Drinking water treatment method: Donors were asked how they treated water for drinking purposes, which included: boiling (boiling water prior to drinking); filtering (filtration of water); chemical treatment (use of water purifier chemicals); or, no treatment (drinking directly from source). Donors with multiple options selected were categorised as multiple methods.
5. Vegetarianism: Donors who ate meat were categorised as non-vegetarian, while others were categorised as vegetarian.
6. Pork consumption: Non-vegetarians who ate pork were categorised as pork consumers and others as pork non-consumers.
7. International travel: Donors who had travelled to other countries were categorised as international travellers, while those who had not, as non-travellers.

### Sample testing

All samples were de-linked prior to testing. Plasma samples were tested individually for HEV IgM (Wantai HEV-IgM ELISA, Beijing Wantai Biological Pharmacy Enterprise Co., Ltd, Beijing, China), as per the manufacturer’s instructions. Any samples testing positive were re-tested in duplicate. Samples that were reactive two or three times were reported as HEV IgM positive. All samples were also tested for HEV-IgG (Wantai HEV IgG ELISA, Beijing Wantai Biological Pharmacy Enterprise Co., Ltd) and HEV antigen (Wantai HEV-Ag^*Plus*^ ELISA, Beijing Wantai Biological Pharmacy Enterprise Co., Ltd) as per the manufacturer’s instructions, using the same testing algorithm as mentioned above for HEV IgM.

### Statistical analysis

Donor data obtained from the questionnaire were entered in to a Microsoft Excel 2010 (Redmond, WA, USA) database. Proportions of donors HEV IgG, IgM or antigen positive were calculated overall and for each of the study variables, and 95% confidence intervals estimated. IBM SPSS Statistics 23 (IBM Centre, NSW, Australia) was used to analyse for statistical inference (chi-square, odds ratio) and to determine association with variables. Some donors did not answer some of the questions on the questionnaire, resulting in missing data for some variables. In these instances, the missing data were subjected to multiple imputations prior to regression analyses. Thus, in the absence of data from donors not responding to the study variables, potential bias in the inference could not be excluded. In addition, donors’ responses to the questions were based on recollection, introducing the risk of possible recall bias.

## Results

Of the 1,845 samples tested, 55 (3.0%, 95% CI 2.2–3.8%) were positive for HEV IgM. The proportion of donors with both HEV IgM and IgG was 2.7%. HEV IgM prevalence was associated with a donor reporting having a history of jaundice or reporting pork consumption (*p* < 0.05) (multivariate analysis) (Table 1). No associations were observed for the other factors investigated (Table 1). Of the donors residing in earthquake affected regions (Kathmandu, Bhaktapur and Kavre), 3.2% (95% CI 2.7–4.0%) of donors were HEV IgM positive, and two donors were positive for HEV antigen. These HEV antigen positive individuals were also HEV IgG positive (Table 2), but negative for HEV IgM. Both HEV antigen positive donors were from Kathmandu and 36 years of age.

**Table 1:** HEV IgM seroprevalence in Nepalese blood donors

Variable	*n* tested	HEV IgM seropositive		Univariate Analysis		Multivariate Analysis	
		*n*	% (95% CI)	Odds ratio (95% CI)	*p* value	Odds ratio (95% CI)	*p* value
Sex							
Female	306	4	1.3 (0.0–2.6)	^a^			
Male	1,539	51	3.3 (2.4–4.2)	2.6 (0.9–7.2)	>0.05		
Age					>0.05		
<25 years	735	18	2.5 (1.3–3.6)	^a^			
25–34 years	682	22	3.2 (1.9–4.6)	1.3 (0.7–2.5)	>0.05		
35–44 years	315	10	3.2 (1.2–5.1)	1.3 (0.6–2.9)	>0.05		
45–54 years	99	5	5.1 (0.7–9.4)	2.1 (0.8–5.8)	>0.05		
55–64 years	14	0	0	0	>0.05		
District					>0.05		
Bhaktapur	135	6	4.4 (1.0–7.9)	7.4 (0.9–61.8)	>0.05		
Kavre	116	3	2.6 (0–5.47)	4.2 (0.4–40.9)	>0.05		
Kathmandu	1,435	45	3.1 (2.2–4.0)	5.12 (0.7–37.4)	>0.05		
Chitwan	159	1	0.6 (0–1.9)	^a^			
Donor status							
Repeat	1,265	40	3.2 (2.2–4.1)	1.2 (0.7–2.2)	>0.05		
First time	580	15	2.6 (1.3–3.9)	^a^			
History of Jaundice							
Yes	212	13	6.1 (2.9–9.4)	2.6 (1.4–4.9)	<0.05	2.5 (1.3–4.7)	<0.05
No	1,633	42	2.5 (1.8–3.3)	^a^			
Family history of jaundice							
Yes	226	8	3.5 (1.1–6.0)	1.3 (0.6–2.7)	>0.05		
No	1,619	47	2.9 (2.1–3.7)	^a^			
Drinking Water Source					>0.05		
Community tap	274	10	3.7 (1.4–5.9)	1.01 (0.3–3.04)	>0.05		
Municipality	940	20	2.1 (1.2–3.1)	0.57 (0.2–1.4)	>0.05		
Others	296	14	4.7 (2.3–7.2)	1.05 (0.2–5.29)	>0.05		
Multiple sources	60	2	3.3 (0–7.9)	1.2 (0.5–3.1)	>0.05		
Underground	250	9	3.6 (1.2–5.9)	^a^			
Drinking water treatment					>0.05		
Boiling	315	13	4.1 (1.9–6.3)	^a^			
Filtering	1,030	30	2.9 (1.9–3.9)	0.7 (0.4–1.5)	>0.05		
Chemical treatment	50	1	2.0 (0–5.9)	0.5 (0.1–3.9)	>0.05		
Multiple methods	202	4	2.0 (0.1–3.9)	0.5 (0.2–1.7)	>0.05		
No treatment	248	7	2.8 (0.8–4.9)	0.7 (0.3–1.8)	>0.05		
Vegetarianism							
Yes	1,663	48	2.9 (2.1–3.7)	^a^			
No	182	7	3.9 (1.1–6.6)	1.4 (0.6–3.2)	>0.05		
Pork consumption							
Yes	700	29	4.1 (2.7–5.6)	1.9 (1.1–3.3)	<0.05	1.8 (1.0–3.2)	<0.05
No	1,145	26	2.3 (1.4–3.1)	^a^			
International travel							
Yes	565	15	2.7 (1.3–4.0)	^a^			
No	1,280	40	3.1 (2.2–4.1)	1.2 (0.6–2.1)	>0.05		

^a^Reference group

**Table 2:** HEV antigen positive blood donors

Variable	Sample 1059	Sample 1303
Collection Date	12/06/2015	13/06/2015
Collection District	Kathmandu	Kathmandu
Age	36	36
Sex	Male	Female
History of Jaundice	Yes	No
Family history of jaundice	No	No
Drinking water source	Municipality	Municipality
Vegetarianism	No	No
Pork Consumption	No	No response
International travel	No	No
HEV IgG	Positive	Positive
HEV IgM	Negative	Negative
HEV antigen	Positive	Positive

HEV IgG was detected in 773 of the 1,845 samples tested (41.9%, 95% CI 39.7–44.2%). The prevalence was significantly higher (*p* < 0.05) in Bhaktapur, Kavre and Kathmandu than the Chitwan district (Table 3). HEV IgG prevalence increased with increasing age and was highest (85.7%) in individuals above 55 years (*p* < 0.05). HEV IgG prevalence was also higher in repeat blood donors, those with a history of jaundice and those reporting pork consumption (*p* < 0.05) (multivariate analysis) (Table 3). Individuals who relied on drinking underground water were associated with having a lower HEV IgG prevalence (*p* < 0.05) (multivariate analysis) (Table 3).

**Table 3:** HEV IgG seroprevalence in Nepalese blood donors

Variable	*n* tested	HEV IgG seropositive		Univariate Analysis		Multivariate Analysis	
		*n*	% (95% CI)	Odds ratio (95% CI)	*p* value	Odds ratio (95% CI)	*p* value
Sex							
Female	306	109	35.6 (30.3–41.0)	^a^		^a^	
Male	1,539	664	43.1 (40.7–45.6)	1.4 (1.1–1.8)	<0.05	1.3 (1.0–1.7)	>0.05
Age					<0.05		<0.05
<25 years	735	155	21.1 (18.1–24.0)	^a^		^a^	
25–34 years	682	330	48.4 (44.6–52.1)	3.5 (2.8–4.4)	<0.05	3.5 (2.7–4.5)	<0.05
35–44 years	315	203	64.4 (59.2–69.7)	6.8 (5.1–9.1)	<0.05	7.6 (5.5–10.5)	<0.05
45–54 years	99	73	73.7 (65.1–82.4)	10.5 (6.5–17.0)	<0.05	10.9 (6.5–18.3)	<0.05
55–64 years	14	12	85.7 (67.4–44.2)	22.5 (5.0–101.4)	<0.05	24.6 (4.9–124.3)	<0.05
District					<0.05		<0.05
Bhaktapur	135	74	54.8 (46.4–63.2)	10.8 (5.9–20.1)	<0.05	13.5 (7.0–26.1)	<0.05
Kavre	116	52	44.8 (35.8–53.9)	7.3 (3.9–13.7)	<0.05	7.0 (3.6–13.8)	<0.05
Kathmandu	1,435	631	44.0 (41.4–46.5)	7.0 (4.1–11.9)	<0.05	8.0 (4.6–14.0)	<0.05
Chitwan	159	16	10.1 (5.4–14.7)	^a^		^a^	
Donor status							
Repeat	1,264	606	47.9 (45.2–50.7)	2.3 (1.8–2.8)	<0.05	1.4 (1.1–1.7)	<0.05
First time	581	167	28.7 (25.1–32.4)	^a^		^a^	
History of Jaundice							
Yes	213	119	55.9 (49.2–62.5)	1.9 (1.4–2.6)	<0.05	2.0 (1.4–2.7)	<0.05
No	1,632	654	40.1 (37.7–42.5)	^a^		^a^	
Family history of jaundice							
Yes	227	114	50.2 (43.7–56.7)	1.47 (1.10–1.97)	<0.05	1.2 (0.9–1.8)	>0.05
No	1,618	659	40.7 (38.3–43.1)	^a^		^a^	
Drinking Water Source					<0.05		<0.05
Community tap	273	103	37.7 (32.0–43.5)	1.2 (0.8–1.7)	>0.05	1.4 (1.0–1.9)	<0.05
Municipality	940	423	45.00 (41.8–48.2)	1.6 (1.2–2.2)	<0.05	0.9 (0.5–1.8)	>0.05
Others	309	135	43.7 (38.2–49.2)	1.3 (0.7–2.3)	>0.05	1.2 (0.8–1.7)	>0.05
Multiple sources	63	25	39.7 (27.6–51.8)	1.5 (1.1–2.2)	<0.05	0.8 (0.6–1.2)	>0.05
Underground	260	87	33.5 (27.7–39.2)	^a^		^a^	
Drinking water treatment					>0.05		
Boiling	315	117	37.1 (31.8–42.5)	^a^			
Filtering	1,030	448	43.5 (40.5–46.5)	1.3 (1.0–1.7)	<0.05		
Chemical treatment	49	18	36.7 (23.2–50.2)	1.0 (0.5–1.9)	>0.05		
Multiple methods	203	91	44.8 (38.0–51.7)	1.4 (1.0–2.0)	>0.05		
No treatment	248	99	39.9 (33.8–46.0)	1.1 (0.8–1.6)	>0.05		
Vegetarianism							
Yes	1,662	701	42.2 (39.8–44.6)	1.1 (0.8–1.6)	>0.05		
No	183	72	39.3 (32.3–46.4)	^a^			
Pork consumption							
Yes	701	323	46.1 (42.4–49.8)	1.3 (1.1–1.6)	<0.05	1.3 (1.0–1.6)	<0.05
No	1,144	450	39.3 (36.5–42.2)	^a^		^a^	
International travel							
Yes	565	280	49.6 (45.4–53.7)	1.6 (1.3–1.9)	<0.05	0.9 (0.7–1.2)	>0.05
No	1,280	493	38.5 (35.9–41.2)	^a^		^a^	

^a^Reference group

## Discussion

HEV outbreaks occur sporadically in developing countries due to faecal contamination of water and poor sanitation [25]. Given a relatively high mortality rate (0.2–4%), which is particularly high in pregnant women (10–25%) [17], HEV is a topic of public health concern in developing countries. Recent devastating earthquakes in Nepal could have facilitated an outbreak of HEV. In this study, we measured previous and current HEV infection in Nepalese blood donors after recent major earthquakes to provide surveillance data on HEV in Nepal and to determine possible risk factors for HEV exposure.

This study was conducted during the months June-September, 2015, after the devastating earthquakes and the monsoon season period, when waterborne outbreaks of HEV were likely to occur [20, 26]. Bhaktapur, Kavre and Kathmandu were among the earthquake-affected districts. In these regions, we report that 3.2% (54/1,686) of the healthy population demonstrated recent HEV exposure (through detection of HEV IgM) and we detected two donors with HEV antigen. The rate of HEV IgM prevalence was lower in the non-earthquake affected region, Chitwan, where a similar pattern was observed for HEV IgG. This suggests less HEV transmission in the Chitwan district. During an epidemic in Biratnagar, Nepal, 2014, HEV IgM prevalence was as high as 94–100% in acute hepatitis patients [11]. In our study, subjects were blood donors, considered healthy and therefore would not capture the symptomatic group of the population. HEV IgM and antigen detection are likely to represent asymptomatic infections in blood donors. Since symptomatic HEV cases are unlikely to be included, studying blood donors may result in an inability to detect the full magnitude of an outbreak. Selecting well donors may additionally result in selection bias of those with pre-existing immunity in hyperendemic areas. However, given that 50% of HEV cases in developing countries are asymptomatic [3], we assume we have identified about half of the cases during this time.

HEV antigen, indicative of current infection, was detected in 2 donors from Kathmandu. Both of these donors were positive for HEV IgG, but negative for HEV IgM. This indicates HEV antigen is likely to persist for a short period, and is undetectable by the time of appearance of HEV IgM. A study has shown that 19% (3/16) of HEV antigen positive samples were HEV IgM negative [27]. Concurrent detection of HEV antigen and IgG in both our donors could indicate re-infection, however, non-specificity of the antigen assay cannot be ruled out. In the absence of HEV RNA testing, the infectious state of antibody and/or antigen positive donors could not be determined.

In the absence of complete population data during epidemic and inter-epidemic periods of HEV circulation, there is no definitive IgM positivity proportion that can be used to define a recent outbreak. The majority of serological studies in epidemics are done in acute cases, and not relevant to background population seroprevalence. However, population serosurveys during known large outbreaks indicate a higher prevalence of IgM positivity than detected in our study. In Sudan in 2012 a serosurvey performed before a large outbreak peak in refugee camps demonstrated an IgM positivity rate of 21.7% [28]. Similarly, in a serosurvey of children aged 0–15 during an Ugandan outbreak, IgM positivity was 37.3% [29]. In endemic areas, asymptomatic positivity in blood donors varies from 0.5 to 5% [30–34]. Our finding of 3.2% IgM positivity in blood donors from earthquake-affected regions is consistent with ongoing endemic transmission. Therefore, we did not find strong evidence of a large post-earthquake HEV outbreak. Persistence of HEV IgG offers protection from subsequent infection; it has been observed that HEV outbreaks occur every few years in India, perhaps corresponding to when herd immunity drops below a threshold [35]. Thus high antibody levels present in the Nepalese population due to previous outbreaks could have provided immunity against re-infection of the exposed population.

HEV outbreaks in Nepal are either focal (where a large number of cases occur over days to weeks in a well-defined small population) or epidemic [9]. This study did not have the power to detect a focal outbreak. The failure of this study to provide evidence of a large HEV outbreak in the months directly following the earthquakes reflects either inability of the study to detect the outbreak, or the absence of a HEV outbreak. It has been estimated that 390,000 individuals left the Kathmandu region immediately following the earthquakes, with movements into the area significantly below normal [36]. These population flows may have decreased the HEV population susceptibility. If migrant populations with lower HEV immunity were disproportionately removed from the at risk population this would decrease the likelihood of an outbreak. The impact of earthquake relief support to public health threats such as provision of clean water and increased awareness of the risk may have also decreased the likelihood of an outbreak. Alternatively, given the lack of baseline HEV IgM positivity in Nepalese blood donors, it may be that 3.2% exposure represents a small outbreak. This argument is strengthened by the prolonged epidemic pattern that typically occurs in Kathmandu and the transient nature of IgM positivity [37]. However, there are no recent published reports on HEV clinical cases that would indicate an outbreak in the general population post-earthquake.

Since HEV in developing countries is commonly associated with drinking contaminated water, there is less awareness of the potential risk of this virus to blood transfusion safety. A retrospective study in India has shown a higher prevalence of HEV infection markers among blood transfusion recipients compared to control groups [38]. HEV is a possible risk to blood supply safety in developed countries [39–41]. For developing countries, however, the main concerns are other modes of transmission, which are the major contributor to the burden of disease. However, HEV can cause chronic infection in immunocompromised individuals [17, 42], and contributes to a higher mortality rate in women during third trimester of pregnancy [3]. Hence, a safe blood supply for these high-risk vulnerable patients should also be of concern in developing countries.

Higher HEV IgG and IgM prevalence was observed in donors who reported eating pork, which is likely an indicator of zoonotic transmission [8]. HEV RNA of unknown genotype and antibodies have been detected in domestic swine in Kathmandu [19]. This suggests zoonotic transmission via consumption of undercooked pork may also contribute to the burden of HEV in Nepal. However, to date, isolation of HEV genotype 3 from humans associated with swine has not been reported in Nepal.

Donors’ responses to drinking water source and method of water treatment were solely based on their preference and their practice of water collection and treatment. Therefore, risk from other sources of water consumed could have also contributed to seropositivity. Based on our observation, lower HEV IgG seroprevalence was associated with individuals relying on an underground water source. A potential explanation could be less likelihood of faecal contamination of underground water compared to other sources. With the drinking water pipelines being adjacent to the sewer system in the Kathmandu district, there is a chance that the drinking water could be contaminated in the event of sewer leakage. Underground water is usually sourced from deep below the earth’s surface and collected directly by manual hand pumps or water pumps and therefore there is a lower possibility of contamination.

HEV IgG prevalence in the blood donor population studied was relatively high. This is in a similar range to previous estimates in Nepal based on population studies [9, 12]. HEV IgG prevalence was highest in Bhaktapur and lowest was in Chitwan, indicating HEV exposure varies between the different regions of Nepal. Geography and other factors, such as water supply systems, in these districts are likely to contribute to these observed differences. HEV IgG prevalence increased with age, which is in agreement with studies in other countries [41, 43], and indicates cumulative exposure. However, this observation differs from previous studies in Nepal, which have shown non-uniform increase with age [9, 12]. The variation is likely to be due to differences in cohort selection between the studies.

## Conclusions

HEV infection in Nepalese blood donors is comparable to the general population. Past exposure to HEV was associated with multiple factors, including age, district of blood collection and consumption of pork. In developing countries like Nepal, where the main transmission route is faecal oral, other modes of transmission including zoonotic and transfusion may also occur. Detection of recent HEV infection in the donor population demonstrates the risk of transfusion-transmission in vulnerable patients in Nepal. Unexpectedly, this study did not provide evidence of a sizeable HEV outbreak after the devastating earthquakes in 2015.

## Abbreviations

CI: Confidence Interval
CBTS: Central Blood Transfusion Service
HEV: Hepatitis E Virus
IgG: Immunoglobulin G
IgM: Immunoglobulin M
NRCS: Nepal Red Cross Society
RNA: Ribonucleic Acid
